# Subcellular protein expression models for microsatellite instability in colorectal adenocarcinoma tissue images

**DOI:** 10.1186/s12859-016-1243-y

**Published:** 2016-10-22

**Authors:** Violeta N. Kovacheva, Nasir M. Rajpoot

**Affiliations:** 1 Department of Systems Biology, University of Warwick, Coventry, CV4 7AL UK; 2 Department of Computer Science, University of Warwick, Coventry, CV4 7AL UK; 3 Department of Computer Science and Engineering, Qatar University, Doha, Qatar; 4 Centre for Molecular Pathology, Institute of Cancer Research, London, SM2 5NG UK; 5 Centre for Evolution and Cancer, Institute of Cancer Research, London, SM2 5NG UK; 6 Division of Molecular Pathology, The Institute of Cancer Research, London, SM2 5NG UK; 7 Department of Pathology, University Hospitals Coventry & Warwickshire NHS Trust, Coventry, CV2 2DX UK

**Keywords:** Multiplex fluorescence imaging, Colorectal tissue architecture, Subcellular protein expression, Protein expression modelling

## Abstract

**Background:**

New bioimaging techniques capable of visualising the co-location of numerous proteins within individual cells have been proposed to study tumour heterogeneity of neighbouring cells within the same tissue specimen. These techniques have highlighted the need to better understand the interplay between proteins in terms of their colocalisation.

**Results:**

We recently proposed a cellular-level model of the healthy and cancerous colonic crypt microenvironments. Here, we extend the model to include detailed models of protein expression to generate synthetic multiplex fluorescence data. As a first step, we present models for various cell organelles learned from real immunofluorescence data from the Human Protein Atlas. Comparison between the distribution of various features obtained from the real and synthetic organelles has shown very good agreement. This has included both features that have been used as part of the model input and ones that have not been explicitly considered. We then develop models for six proteins which are important colorectal cancer biomarkers and are associated with microsatellite instability, namely MLH1, PMS2, MSH2, MSH6, P53 and PTEN. The protein models include their complex expression patterns and which cell phenotypes express them. The models have been validated by comparing distributions of real and synthesised parameters and by application of frameworks for analysing multiplex immunofluorescence image data.

**Conclusions:**

The six proteins have been chosen as a case study to illustrate how the model can be used to generate synthetic multiplex immunofluorescence data. Further proteins could be included within the model in a similar manner to enable the study of a larger set of proteins of interest and their interactions. To the best of our knowledge, this is the first model for expression of multiple proteins in anatomically intact tissue, rather than within cells in culture.

## Background

Recent popularity of multiplex immunofluorescence (IF) imaging is generating massive amounts of digital image data. In consequence, the demand for development of robust analytical methods for quantitative analysis of the image data is on the rise. Realistic synthetic data could provide an objective way of validating and comparing such methods. Building accurate protein expression models requires taking into account their spatial distributions since the subcellular location of a protein is so critical to its function that the same protein can have different functions at different locations [1]. The Virtual Cell project [2] enables the formulation of both compartmental and spatial partial differential equation models. Similarly, Monte Carlo Cell (MCell) and Smoldyn [3, 4] use agent-based methods which simulate each molecule individually and evaluate their diffusion and probability of interactions on a per-particle basis for each time step. Although computationally extremely expensive, these methods have high spatial resolution and are successful at modelling interactions of small numbers of heterogeneously distributed molecules.

While the above methods can be useful for studying the dynamics of protein interaction, they do little to model the microscopic level cell structure, which is necessary for validation of image analysis methods such as cell-compartment classification methods [5–9]. To address this issue, Zhao and Murphy [10] presented a machine learning method to generate realistic cells with labelled nuclei, membranes and a protein expressed in a cell organelle. Parameters for these models were learned from real images of cells in culture. However, these generative models are restricted to individual cells in culture and only one protein of interest at a time. Hence, they do not capture the dynamic interplay between important proteins localised in certain cell compartments in anatomically intact tissue as opposed to freely moving cells in culture.

Other frameworks for generating synthetic IF data include the SIMCEP simulator, which can simulate large 2D cell populations with realistically looking cytoplasm, nuclei and cell organelle [11]. Svoboda et al. [12] generated a model to fully simulate 3D image data of cell nuclei, with realistic distribution [13], and later of healthy colon tissue [14]. However, these models only include cell nuclei. In addition, the shape of the nuclei in the colon tissue model of [14] is not very realistic due to the presence of sharp corners generated from Voronoi diagrams and does not reflect the variety of cell phenotypes found in real tissue. Heterogeneous cell populations expressing different protein markers can be simulated using the SimuCell toolbox [15]. The first method for simulating bright-field microscopy was proposed for synthesising cervical smears [16]. However, tissue microenvironment was not taken into account in that work. More recently, a model has been proposed for simulating the microenvironment of healthy and cancerous colon tissue [17, 18]. This model has a number of user-defined parameters that allow control over the tissue appearance and is capable of simulating microscopy images for cancers of various differentiation grade.

**Fig. 1:**
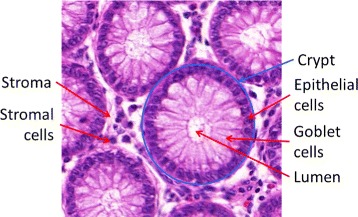
A Hematoxilyn and Eosin (H&E) image depicting the structure of healthy colon tissue

Healthy colon tissue microenvironment is composed of a single layer of epithelium forming glandular structures, called crypts (as shown in Fig. 1). The crypts consist mostly of three types of cells: epithelial (absorptive) cells, goblet cells, and stem cells (Fig. 1), and extend down to sit on the *muscularis mucosae*. Stroma fills the space between the crypts and contains several types of cells, such as lymphocytes, plasma cells and fibroblasts. As the colorectal adenocarcinoma (CRA) develops from normal tissue, the epithelium exhibits increased dysplasia (pre-malignant change with disordered growth and mutation) and there are fewer mucus-containing goblet cells, reflecting a lack of normal cellular differentiation. CRA is a heterogeneous group of diseases which have distinctive genetic and epigenetic basis [19]. It arises following one of the three pathways: microsatellite instability (MSI), chromosomal instability (CIN) or CpG island methylator phenotype (CIMP) pathways. The CIN pathway is the most common and is characterised by widespread imbalances in chromosome number and loss of heterozygosity (loss of an entire gene). It can result from accumulation of mutations in specific tumour suppressor genes and oncogenes that activate pathways critical for CRA such as chromosomal segregation, telomere stability, and the DNA damage response [20]. On the other hand, epigenetic instability is now believed to be implicated in the pathogenesis of almost one third of colorectal cancers [21]. CRAs with CIMP are characterised by epigenetic loss of function of tumour suppressor genes without mutations [21, 22]. The MSI pathway is discussed in more detail below.

Microsatellites are simple repeat sequences of 1 to 6 base pairs (also known as short tandem repeats) and are particularly prone to replication errors. Defects in one of the four DNA mismatch repair (MMR) genes (MLH1, MSH2, MSH6, PMS2) cause small changes in the number of repeats of microsatellites throughout the genome, consequently resulting in the development of the MSI. Mismatch repair is a complex process that depends on the MMR proteins and multiple proteins that interact directly with the DNA [23]. The MSH2 and MSH6 proteins exist as a heterodimer, which forms a sliding clamp on the DNA strand. When MSH2 recognises a DNA base pair mismatch, it recruits the MLH1-PMS2 heterodimer. Repairing the mismatch requires coordinated activity of DNA repair proteins and the precise mechanisms are still under investigation [24, 25].

Around 15 % of CRAs are characterised by a high degree of MSI (MSI-high) [24], and of these, about 1 in 5 (3–5 % overall, [26]) are due to Lynch syndrome (LS), previously known as hereditary nonpolyposis colorectal cancer (HNPCC). LS is the most common inherited CRA syndrome and it predisposes the patient to cancers of multiple organ systems, including the gastrointestinal tract. It is important to identify patients with LS as it allows for increased surveillance of the affected individual and of potentially affected family members. Hence, preliminary screening is often done using IHC to detect MSI. Most MSI-high CRAs are caused by epigenetic silencing of the MLH1 gene (≈50 %) or the MSH2 gene (≈40 %) [27]. Mutations in MSH6 and PMS2 occur only in about 10 % of LS patients [28, 29]. In addition, Samowitz et al. [30] considered the relationship between P53 mutations and MSI in CRAs. The study considered mutation in the P53 gene to be indicated by over-expression (over 50 % of tumour cells expressing) of the protein in immunohistochemistry (IHC) data. They found that P53 over-expression occurred in 56 % of microsatellite stable tumours and only 20 % of unstable tumours.

In this work, we propose models for subcellular expression of proteins associated with MSI, namely MLH1, MSH2, MSH6 and PMS2, and tumour suppressor proteins, such as P53 and PTEN. These proteins were selected as a case study to illustrate how a variety of proteins can be included within the framework to enable the generation of synthetic multiplex fluorescence image data of both healthy and cancerous tissue samples. The models have been integrated within a model of the tumour microenvironment of colorectal cancer [17, 18], and take into account the cell phenotypes present in the tissue as well as the presence of relevant gene mutations. We have also developed models for a number of subcellular organelles, which were learned from real high-resolution confocal microscopy images. We have validated the models by comparing the distributions of morphological features of the cell organelles, by performing combinatorial molecular phenotype analysis, and by constructing the cell-level protein co-localisation networks. The analysis has demonstrated that the model generates realistic image data which could be used to validate and compare various image analysis methods such as cell-compartment classification methods, frameworks for studying protein co-localisation or protein expression grading.

To the best of our knowledge, this is the first model for expression of multiple proteins in anatomically intact tissue, rather than within cells in culture.

## Methods

It is important to study tumour heterogeneity and the MSI, as they could guide treatment and help diagnose Lynch syndrome. We have considered the four MMR proteins (MLH1, PMS2, MSH2, MSH6). Mutations in genes producing these proteins are the cause for MSI. In addition, we consider P53 which has been found to be also associated with the condition [30] and PTEN which is an important CRA biomarker. These proteins have varied subcellular expression patterns (Table 1) and provide an interesting case study demonstrating how several different protein expressions could be included within the proposed model.

**Table 1:** Details of the subcellular location of proteins obtained from the Human Protein Atlas (HPA) [31]

Protein	Subcellular location
MLH1	Nucleoli, weak expression in the nucleus and cytoplasm
PMS2	Nucleus but not nucleoli, weak expression in cytoplasm
MSH2	Nucleus but not nucleoli, vesicles
MSH6	Mainly in the nucleus but not nucleoli. In addition localised to the cytoplasm, golgi apparatus & vesicles.
P53	Nucleus but not nucleoli
PTEN	Nucleus but not nucleoli and in the cytoplasm

In order to model the expression of proteins, we first need to have models for the cell organelles where the proteins of interest are expressed. These are detailed in Table 1. We use real confocal IF data from the Human Protein Atlas (HPA, http://proteinatlas.org) [31] to learn features of the organelles that can then be incorporated into the model. The IF images of cultured cells are utilised instead of the IHC images of CRA since the latter do not provide high enough resolution to consider the subcellular protein expression patterns. Once we have realistic models for the cell organelles, we then develop models for the proteins based on where they are expressed and under what conditions. Details of this process are given below.

### Learning from the real data

We have utilised high resolution IF images of cultured cells from the HPA [31] for learning parameters for our model. In order to model the proteins of interest, we need to develop models for the nucleoli, golgi apparatus and the vesicles. For each organelle, we have used proteins known to be highly specific to that organelle. To obtain sufficient data, we have used 2 or 3 proteins for each cell organelle, as detailed in Table 2. For each cell organelle, we consider a total of 10 images split nearly evenly between the proteins, with the number of images used for each protein depending on how many good quality images are available.

**Table 2:** Proteins tags used for modelling cell organelles

	Cell organelle		Protein tags	
	Nucleoli		MLH1 & RRP1B	
	Golgi		GOLGA2 & GORASP2	
	Vesicles		ABCD3, PSAP & PECR	

**Fig. 2:**
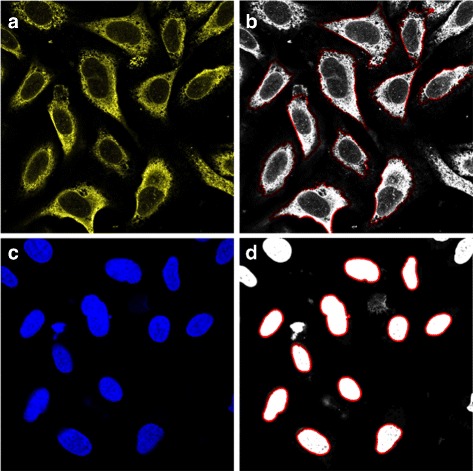
Examples of cell and nuclear segmentation. **a** and **c** show the original ER and nuclear channels. **b** and **d** show the segmentation borders in red

In order to learn from the real IF data, we first need to segment the individual cells, nuclei and cell organelles. Cell and nuclear segmentation was performed using the seeded watershed segmentation method proposed in [32]. The procedure involves thresholding the DAPI image with the threshold being determined as the intensity of the most common pixel. Next, the binary nuclear image is eroded and small objects and objects touching the boundary of the image are removed. This way we could ensure that the seeds generated were not as a result of noise in the image. Nuclei with diameter outside the range of [5, 20] *μ*m are considered erroneous seeds and are also removed. For cell segmentation, the endoplasmic reticulum (ER) channel was used to determine background seeds from large areas of pixels with zero intensity. The seed locations and the inverted ER image are then used in a seeded watershed algorithm, which is a segmentation algorithm identifying “catchment basins” and “watershed ridge lines” in an image by treating it as a surface where light pixels are high and dark pixels are low. For nuclear segmentation, the nuclear (DAPI) channel was used to determine both foreground and background seeds. Examples of the results are shown in Fig. 2.

**Fig. 3:**
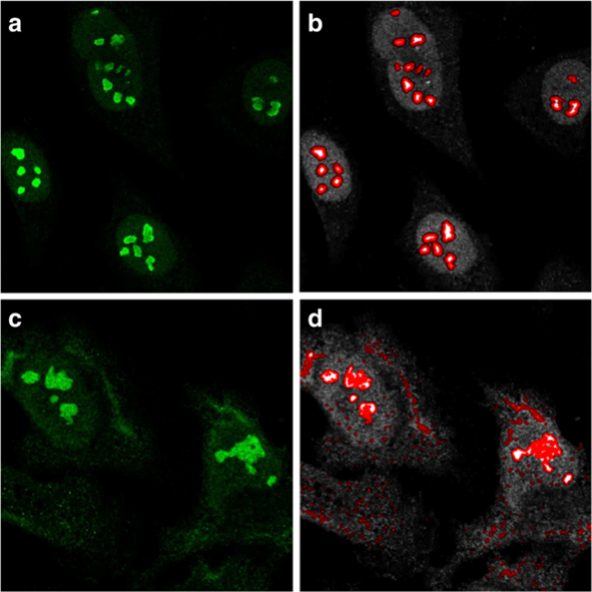
Examples of nucleoli segmentation. **a** and **c** show the original channels for MLH1 and RRP1B images, respectively. **b** shows segmentation results from the seeded watershed segmentation method with borders shown in red. **d** shows segmentation results from the adaptive thresholding method

For the purpose of segmenting the cell nucleoli, a single channel showing a relevant antibody was thresholded to remove background noise and used to obtain both background and foreground seeds. The same segmentation as above was then followed. Similarly to above, nucleoli with diameter outside the range of [0.5, 3] *μ*m are removed. The results are shown in Fig. 3.

**Fig. 4:**
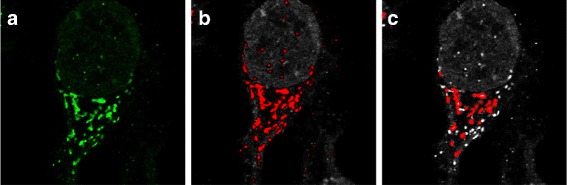
Examples of golgi segmentation. **a** shows the original channel for a GOLGA2 image. **b** shows segmentation results from the adaptive thresholding method with borders shown in red. **c** shows segmentation results from the seeded watershed segmentation method

**Fig. 5:**
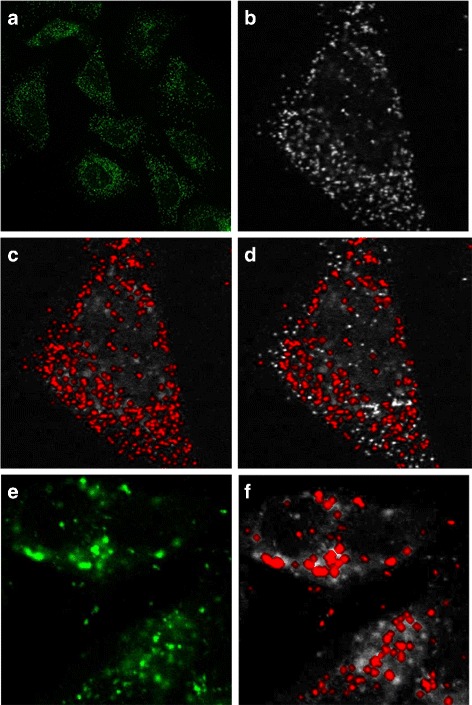
Examples of vesicles segmentation. **a** shows the original channel for an ABCD3 image. **b** and **e** show enlarged sections of the original channels for ABCD3 and PSAP images, respectively. **c** and **f** show segmentation results from the adaptive thresholding method with borders shown in red. **d** shows segmentation results from the seeded watershed segmentation method

When segmenting the vesicles and golgi apparatus, the above method didn’t perform satisfactorily due to the small size of the objects and the high level of noise in the images (Figs. 4 c and 5 d). For this reason, we have instead used an adaptive mean filter to highlight image features and then Otsu threshold to segment the image (Figs. 4 and 5). Objects containing less than 5 pixels were considered noise and were discarded. Thresholding is unable to separate touching organelles. However, this issue would persist even with more sophisticated algorithms as it is due to the fact that the pixel resolution is not high enough to enable one to see whether a large object is a single large organelle or if it consists of two closely located vesicles. We can see that the method performs very well even at high levels of noise (Fig. 5 f). On the other hand, this method tends to over-segment the nucleoli and produces many false positives (Fig. 3 d). The segmentation procedure resulted in 484 nucleoli from 86 cells, 3433 golgi objects from 83 cells and 12,764 vesicles from 72 cells being identified.

**Fig. 6:**
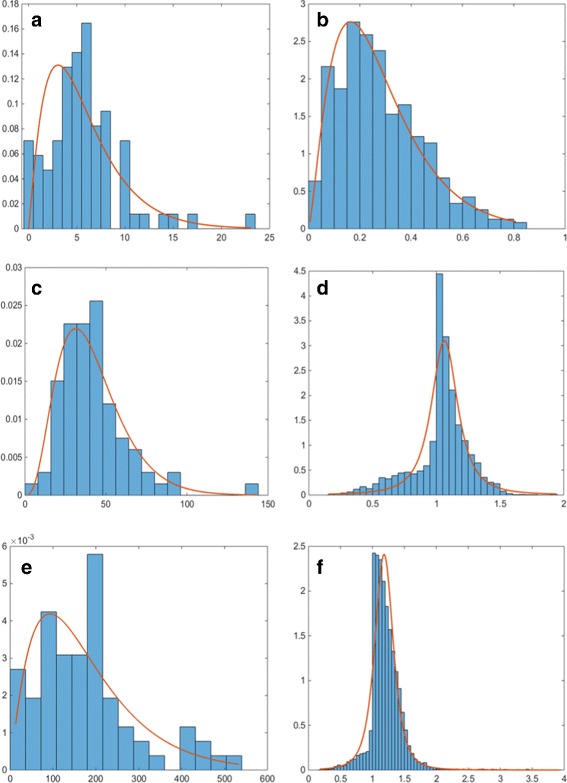
Estimated probability distribution functions for the number (*left column*) and position (*right column*) of the **a**, **b** nucleoli, **c**, **d** golgi and **e**, **f** vesicles

**Fig. 7:**
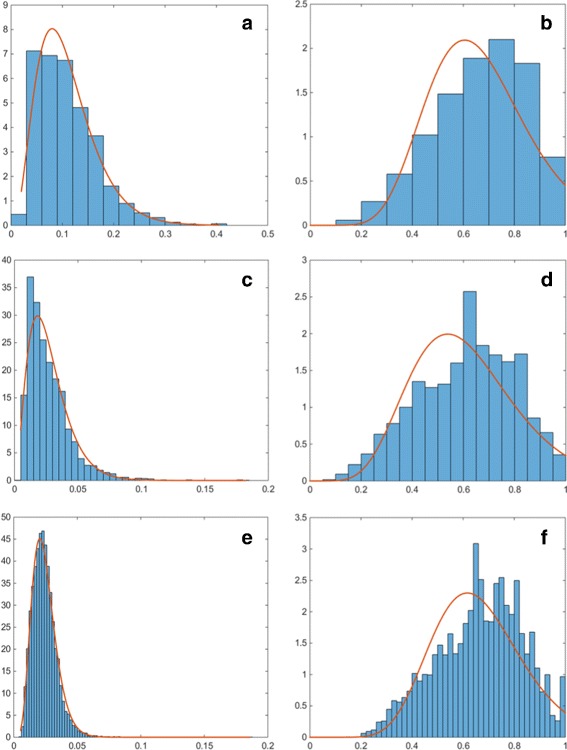
Estimated probability distribution functions for the ratios between the minor axes of the organelles and the nucleus of the corresponding cell (*left column*) and between the minor and major axes of the organelles. Figures show the ratios for **a**, **b** nucleoli, **c**, **d** golgi and **e**, **f** vesicles

Once we have all the objects segmented, we can extract morphological features representing the cell organelles to be incorporated into the model. We extract several features describing the organelles and their distribution within the cell. For each of them, we use maximum likelihood estimation to estimate a probability distribution function (PDF) which is incorporated into the model. Firstly, we obtain the numbers of organelles within each segmented cell. These are modelled using a Gamma PDF, as this distribution provided the best fit, and the results are shown in Fig. 6 (left column). We then consider the size and shape of the organelles. Since the real data available is for different types of cultured cells, instead of estimating the size of the cell organelles directly, we consider the ratio between the minor axes of the cell organelle and that of the corresponding nucleus. We assume that the shape of the cell nucleus is approximately the same in tissue and in cell culture. The distributions of this ratio and the estimated Gamma PDFs for each cell organelle are shown in Fig. 7 (left column). This ratio generalises better to cells in a tissue and at different magnifications. To estimate the shape of the organelles, we consider the ratio between the minor and major axes of the segmented objects. The distributions of this feature and the estimated Gamma PDFs for each cell organelle are shown in Fig. 7 (right column).

**Fig. 8:**
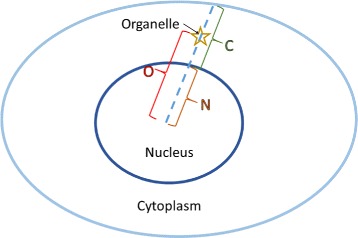
Diagram for calculating the position feature. The star marks the position of the organelle of interest

The last feature considered is the relative position of the organelle within the cell. We considered the line from the centre of the cell nucleus going through the centre of the organelle of interest. Let the distance between the centre of the nucleus and the point where the line crosses the nuclear membrane be given by *N*. Let the distance between the centres of the nucleus and the organelle be given by *O*, and the distance between the points where the line crosses the nuclear and plasma membranes be given by *C* (as shown in Fig. 8). Then, the distance feature is given by

1 $$  D= 1- \frac{N-O}{N+C}.  $$

Consequently, the minimum value of *D*=1−*N*/(*N*+*C*) means the organelle is located at the centre of the nucleus and as *D*→1 the cell compartment is located closer to the nuclear membrane but within the nuclear boundary. A value of *D*>1 describes an organelle that is outside the nuclear boundary and the distance from it is given proportionate to the distance between the centre of the nucleus and the cell membrane. The distributions of this feature and the estimated PDFs for each cell organelle are shown in Fig. 6 (right column). The distribution of the nucleoli position was well estimated by a Gamma PDF. On the other hand, most of the vesicles and golgi objects were found close to the nuclear membrane and so a *t* Location-Scale distribution gave a better fit.

### Modelling cell organelles

**Fig. 9:**
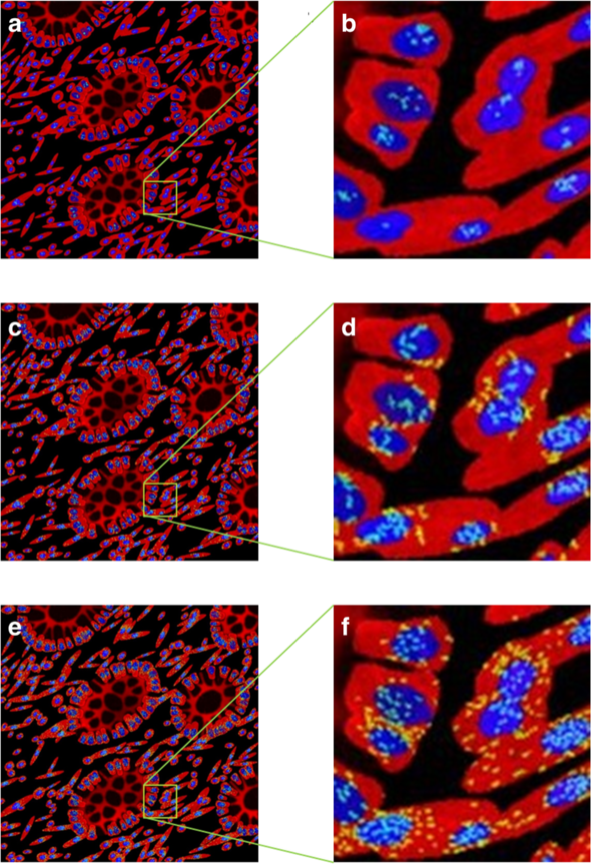
Examples of generated cell organelles. In all images the cytoplasm is shown in red, nuclei in blue and the green channel shows **a**, **b** the nucleoli, **c**, **d** the golgi and **e**, **f** the vesicles. **b**, **d**, **f** show close-up sections of **a**, **c**, **e**, respectively, with the section identified by the green square

For modelling the different cell compartments, we use the deformed circle model used in Kovacheva et al. [18]. When we are generating cell organelles of a particular type, we draw model parameter values from the relevant PDFs as described above. However, we also impose certain restrictions on the parameter values based on the size of the cell in consideration. For each cell, first we choose the number of organelles to be created. We only place a new cell organelle if that type of organelles are not taking up more than 12 or 18 % of the cell area for golgi and vesicles, respectively, and 20 % of the nuclear area for nucleoli. These constraints were set up to address the fact that other parameter values are drawn independently and so may result in unrealistic examples where a large number of organelles with relatively great size are generated. The values were set based on observations from the real data where golgi and vesicles took up to 4 and 6 % of the cell area, and the nucleoli took up to 19.3 % of the nucleus. The first two values were scaled up as the cytoplasm of cells in a tissue has more compact shape and so the 2D projection of it would give a much smaller area. On the other hand, we don’t expect the nucleus to significantly change shape and so the threshold was held nearly the same. For each cell organelle to be placed, we choose the length of the minor axis by drawing a value for the ratio between the nuclear minor axis and that of the organelle. A minimum length of 1 pixel is set. To determine the length of the major axis, we draw a value from the PDF estimated for the ratio between the minor and major organelle axes. Finally, we need to estimate the position of the organelle. For this, we draw a value from the PDF of the distance feature and select the direction from the nuclear centre at random. Using (1), we can then estimate the distance from the nuclear centre. The resulting organelles are shown in Fig. 9.

### Modelling protein expression

With a view to include an IF channel per protein marker into the model, three user-defined parameters were introduced per protein. These define whether or not the protein has been imaged, whether there is a mutation in the gene, and what fraction of the epithelial cells express the protein. Six proteins were included in the model, namely MLH1, PMS2, MSH2, MSH6, P53 and PTEN. The protein expression within each organelle is generated using a well-known procedural model [33] for texture synthesis. Details of each are given below. In addition, the user could choose to produce samples that are representative of the population. In that case, the model would include an MMR protein mutation with a 15 % probability. If a mutation occurs, it has a probability of 50 % of being in the MLH1 gene, 40 % in MSH2, 7 % in MSH6, and 3 % in PMS2 [27]. In cases without mutation, P53 has 50 % probability of being overexpressed in epithelial cells, whereas in MSI cases it is overexpressed in only 20 % of the cases [30].

**Fig. 10:**
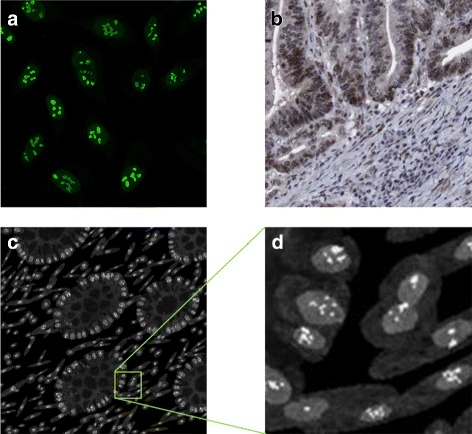
Modelling MLH1. **a** subcellular location of MLH1 in cultured cells imaged using a confocal fluorescence microscope, **b** MLH1 expression in a histology image of CRA; Images **a**, **b** are from the HPA. **c**, **d** synthetic images for MLH1 with **d** a scaled up sections from (**c**). Images are from the same sample as shown in Fig. 9. In this simulation all cells are expressing the protein

The subcellular expression for MLH1 was modelled as described in Table 1 and shown from confocal fluorescence images of cultured cells in Fig. 10 a, namely the protein has a strong expression in the nucleoli and weak expression in the rest of the nucleus. We can see that this also agrees with what is observed when the cells are in a tissue (Fig. 10 b). If the user specifies a mutation in the MLH1 gene, the protein is not expressed in the epithelial cells. Otherwise, the user can specify what fraction of the epithelial cells are expressing the protein. It is worth noting that, in practice, even if only a small fraction of epithelial cells express the MMR proteins, the sample is graded as positively stained. Most stromal cells would always express the MMR proteins and, in the clinic, this serves the pathologists as a positive control that the tissue has been stained. Within the model, all stromal cells would always express MLH1. Examples of IF protein marker images generated are shown in Fig. 10 c, d.

**Fig. 11:**
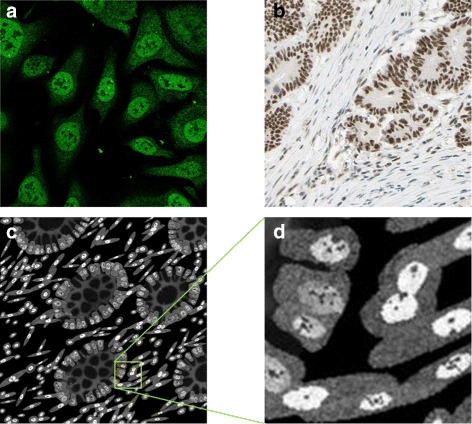
Modelling PMS2. **a** subcellular location of PMS2 in cultured cells imaged using a confocal fluorescence microscope, **b** PMS2 expression in a histology image of CRA; Images **a**, **b** are from the HPA. **c**, **d** synthetic images for PMS2 with **d** a scaled up sections from (**c**). Images are from the same sample as shown in Fig. 9

The real confocal fluorescence images from HPA showed strong expression of PMS2 in the nucleus excluding the nucleoli and weak expression in the cytoplasm (Fig. 11 a). We can see that this also agrees with hat is observed when the cells are in a tissue (Fig. 11 b). If the user specifies a mutation in the PMS2 gene, the protein is not expressed in the epithelial cells. In addition, the same limited expression would occur if there is a mutation in the MLH1 gene as the two are binding partners (Table 3). Otherwise, the user can specify what fraction of the epithelial cells are expressing the protein and these are taken to be a subset of the epithelial cells expressing MLH1. As above, all stromal cell would always express PMS2. Example of synthesised MLH1 protein images are shown in Fig. 11 c, d.

**Table 3:** Effects of mutations in the MMR genes on protein expression in epithelial cells

	Defective gene		Imaging results	
	MLH1		Loss of MLH1, PMS2	
	PMS2		Isolated Loss of PMS2	
	MSH2		Loss of MSH2, MSH6	
	MSH6		Isolated Loss of MSH6	

**Fig. 12:**
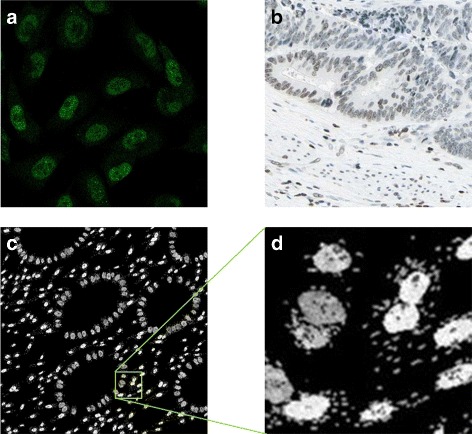
Modelling MSH2. **a** subcellular location of MSH2 in cultured cells imaged using a confocal fluorescence microscope, **b** MSH2 expression in a histology image of CRA; **a**, **b** are from the HPA. **c**, **d** synthetic images for MSH2 with (**d**) a scaled up sections from (**c**). Images are from the same sample as shown in Fig. 9

The subcellular expression for MSH2 was modelled as described in Table 1 and seen in confocal IF images of cultured cells from HPA as shown in Fig. 12 a, namely the protein has a strong expression in the nucleus and weak expression in the nucleoli. The same expression pattern is observed when the cells are in tissue (Fig. 12 b). To generate a realistic texture for this protein we use the chromatin texture used for the nuclear channel of the THeCoT model [18]. If the user specifies a mutation in the MSH2 gene, the protein is not expressed in the epithelial cells. Otherwise, the user can specify the fraction of the epithelial cells expressing the protein. All stromal cells would always express the molecule. Example of synthetic MSH2 protein images are shown in Fig. 12 c, d.

**Fig. 13:**
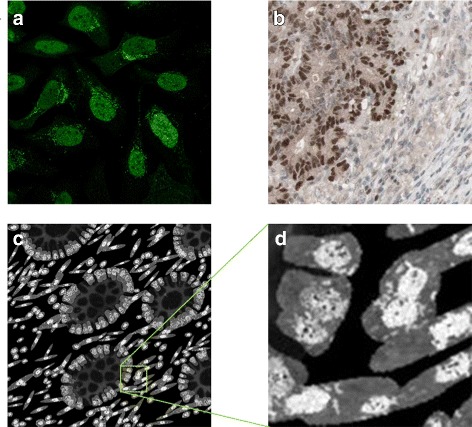
Modelling MSH6. **a** subcellular location of MSH6 in cultured cells imaged using a confocal fluorescence microscope, **b** MSH6 expression in a histology image of CRA; Images **a**, **b** are from the HPA. **c**, **d** synthetic images for MSH6 with (**d**) a scaled up sections from (**c**). Images are from the same sample as shown in Fig. 9

Both in vivo and in vitro cells have strong expression of MSH6 in the nucleus excluding the nucleoli, the vesicles and golgi apparatus, and weak expression in the cytoplasm (Table 1, Fig. 13 a, b). If the user specifies a mutation in the MSH2 or MSH6 genes, the protein is not expressed in the epithelial cells (Table 3). Otherwise, the user can specify what fraction of the epithelial cells are expressing the protein and these are taken to be a subset of the epithelial cells expressing MSH2. As above, all stromal cells would always express MSH6. Example of synthetic MSH6 protein images are shown in Fig. 13 c, d.

**Fig. 14:**
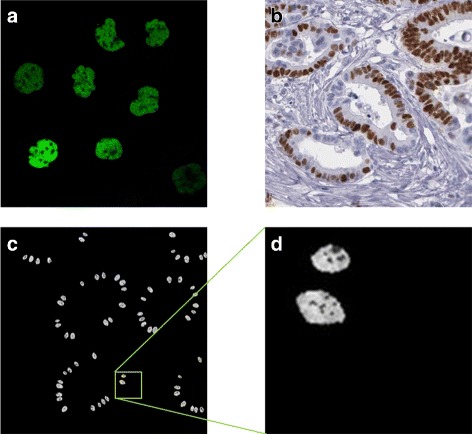
Modelling P53. **a** subcellular location of P53 in cultured cells imaged using a confocal fluorescence microscope, **b** P53 expression in a histology image of CRA; **a**, **b** are from the HPA. **c**, **d** synthetic images for P53 with (**d**) a scaled up sections from (**c**). Images are from the same sample as shown in Fig. 9

P53 has a strong expression in the nucleus excluding the nucleoli (Table 1, Fig. 14 a, b). Unlike the MMR genes, P53 is not expressed in the stromal cells. Hence, to avoid a blank image in the stack, the model assumes that there is some expression of the protein in the epithelial cells. The user can specify what fractions of the epithelial cells are expressing the protein. Example of synthetic P53 protein images are shown in Fig. 14 c, d.

**Fig. 15:**
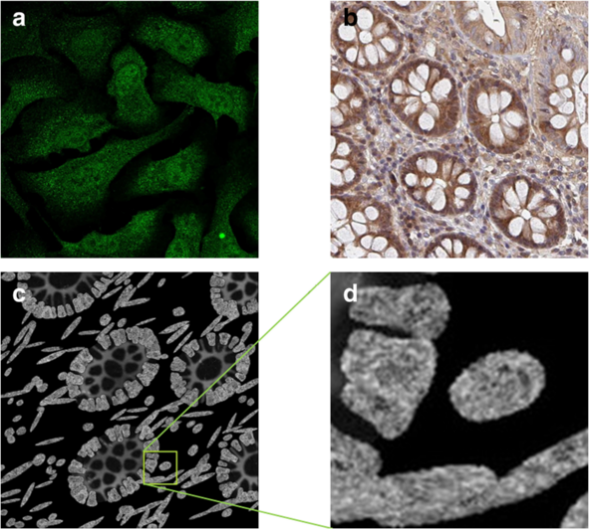
Modelling PTEN. **a** subcellular location of PTEN in cultured cells imaged using a confocal fluorescence microscope, **b** PTEN expression in a histology image of CRA; **a**, **b** are from the HPA. **c**, **d** synthetic images for PTEN with (**d**) a scaled up sections from (**c**). Images are from the same sample as shown in Fig. 9

Similarly, PTEN expression is modelled within the nucleus but not the nucleoli or in the cytoplasm as shown in Fig. 14 d. Unlike P53, PTEN is expressed in some stromal cells. The fraction of stromal cells expressing it is chosen at random to be between 30 and 70 %, based on observations from the real data, an example of which is shown in Fig. 15 b. A sample image showing expression pattern of this protein marker is shown in Fig. 15.

## Results and discussion

We have focussed on six proteins associated with MSI in colorectal cancer. These are commonly screened for in clinical practice and developing the protein expression models could aid the development of frameworks for automatic grading. The user could choose to have a sample that is generated with the probability of mutation representative of the general population. In this case, they also need to specify which of the six proteins they wish to be included in the resulting images. Alternatively, they can specify where the mutation occurs. The model takes into account dependencies of binding pairs of the MMR proteins, and hence, if a mutation occurs in MLH1 or MSH2, its binding partner would also have inhibited expression in epithelial cells. Each protein subcellular expression pattern mimics the behaviour observed in real high-resolution IF data. In this way, we can capture protein co-localisation patterns. In addition, developing realistic protein expression models could potentially aid the discovery of yet unknown protein interactions.

### Subcellular Organelle Features

**Fig. 16:**
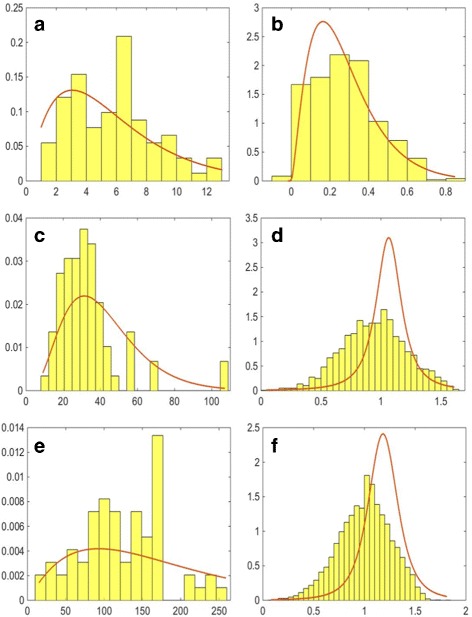
Probability distributions for the synthesised number (*left column*) and position (*right column*) of the **a**, **b** nucleoli, **c**, **d** golgi and **e**, **f** vesicles. The probability distribution functions shown are the ones estimated for the real data, shown in Fig. 6

**Fig. 17:**
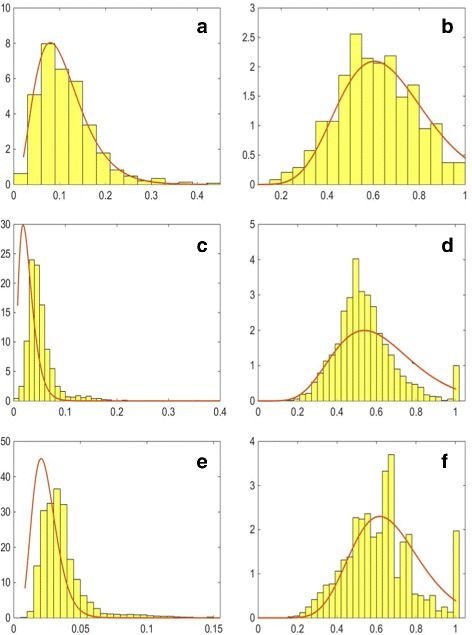
Probability distributions for the ratios between the minor axes of the synthesised organelles and the nucleus of the corresponding cell (*left column*) and between the minor and major axes of the synthesised organelles (*right column*). Figures show the ratios for **a**, **b** nucleoli, **c**, **d** golgi and **e**, **f** vesicles. The probability distribution functions shown are the ones estimated for the real data, shown in Fig. 7

**Fig. 18:**
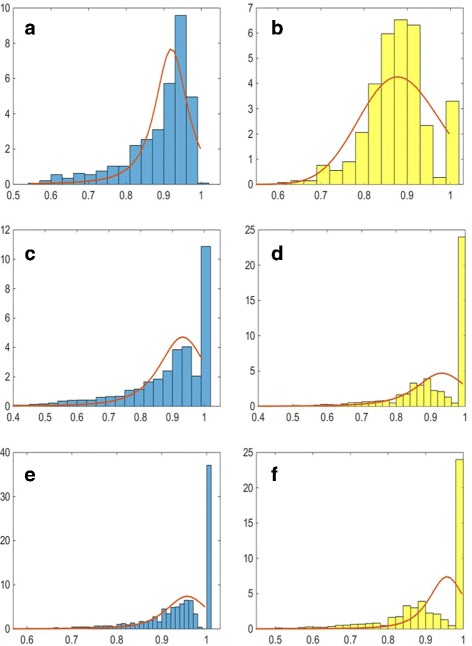
Probability distributions for the real (*left column*) and synthesised (*right column*) solidity of the **a**, **b** nucleoli, **c**, **d** golgi and **e**, **f** vesicles. The probability distribution functions shown are the ones estimated for the real data

**Fig. 19:**
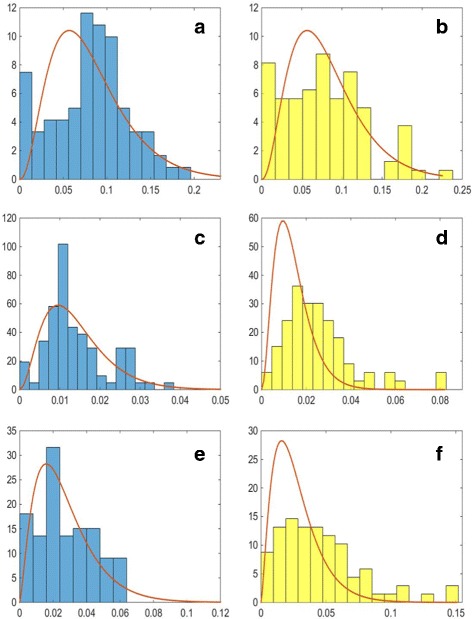
Probability distributions for the cell area fraction taken up by the real (*left column*) and synthesised (*right column*) organelles. **a**, **b** show the fraction of nuclear area taken up by the nucleoli. The fraction of cytoplasmic area taken up by (**c**, **d**) golgi and (**e**, **f**) vesicles is also considered. The probability distribution functions shown are the ones estimated for the real data

In order to assess the quality of the spatial protein expression models, we assess how well the cell organelles have been modelled. We consider how accurately organelle features that have been used as input to the model have been generated within the synthesised data. In order to perform the comparison, we generated 10 well-differentiated samples with the same magnification and image resolution as the real images. This resulted in the generation of 8,663 vesicles, 2,336 golgi and 394 nucleoli. Comparison of the histograms has been been performed using the Kullback-Leibler divergence between the real and synthetic distributions. The results are shown in Table 4. The distributions of the numbers of organelles per cell and their position are shown in Fig. 16. We can see that the distributions of the numbers of organelles are reasonably good approximations of the real PDFs. For the number of golgi, we can see that there are a small number of cells with a very high number of golgi organelles. However, a similar, although smaller peak in the histogram can be observed in the real data (Fig. 6 c). On the other hand, we can see a wider distributions for the position parameter of the synthesised golgi and vesicles. This is due to the fact that when the position of these organelles is being calculated, the method assumes that the nucleus is in the centre of the cell, rather than displaced towards the base of the cell. Hence, the problem does not occur in stromal cells and high-grade cancer samples. On the other hand, the distributions for the ratio between the minor axes of the synthesised organelles and the nucleus of the corresponding cell as shown in Fig. 17 (left column) and between the minor and major axes of the synthesised organelles in Fig. 17 (right column) show very good agreement with the PDFs estimated from the real data. We have also considered features that have not been explicitly learned from the real data. Figure 18 shows the distributions of the solidity for real and synthesised organelles and we can observe very good agreement between the two. In Fig. 19 we consider the area taken up by the organelles. Figure 19 a, b shows the fraction of the nucleus taken by the nucleoli. Figure 19 c–d illustrates the fraction of the total area of the cell taken by the golgi and vesicles. Although the area of the organelles is not specified explicitly within the model, we observe good agreement between the real and synthesised distributions.

**Table 4:** Kullback-Leibler divergence between real and synthetic distributions of features

	Number	Position	Organelle/	Organelle	Solidity	Area
			nucleus ratio	axes ratio	fraction	
Nucleoli	0.06	0.05	0.01	0.07	0.25	0.24
Golgi	0.26	0.24	0.76	0.36	0.68	0.33
Vesicles	0.35	0.41	0.48	0.17	0.08	0.17

### Combinatorial molecular phenotypes

**Fig. 20:**
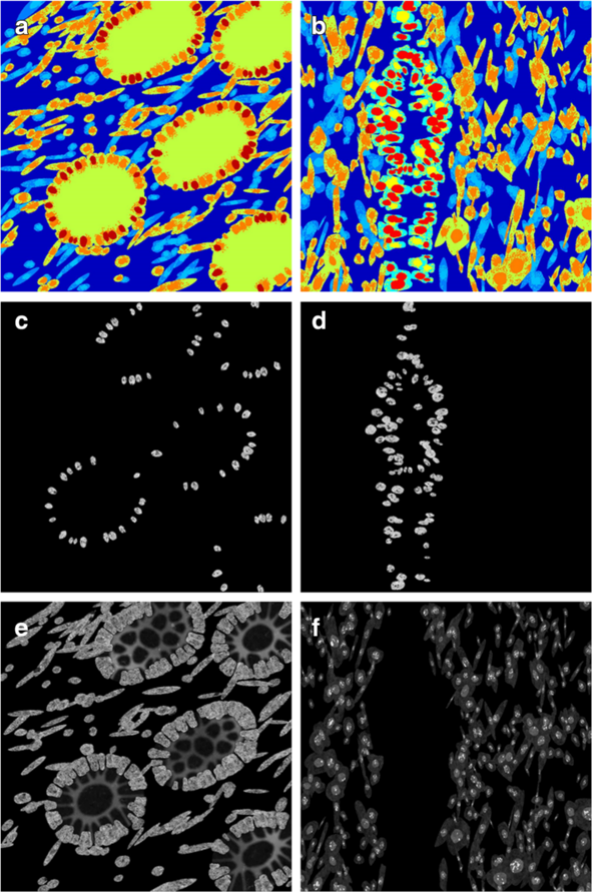
Combinatorial molecular phenotype analysis. **a** and **b** show the CMPs obtained for healthy and cancerous samples, respectively. **c** and **d** show expression of P53 in healthy and cancerous samples, respectively. **e** shows the expression of PTEN in a healthy sample. **f** shows expression of MLH1 in a cancer sample

One imaging technique allowing acquisition of multiplex IF images is the Toponome Imaging System (TIS) [34]. One way of analysing such data is to threshold all the channels, obtaining at each pixel a 0 where the protein is absent and 1 where it is present. Then, the protein expression signals can be expressed as a binary code called Combinatorial Molecular Phenotype (CMP) [34, 35]. We have performed this analysis to compare the CMPs found in a healthy sample and a moderately differentiated sample with a mutation in the MLH1 protein. The healthy sample contained a total of 389 cells, whereas the cancerous sample contained 455 cells. The results are shown in Fig. 20. We can see that the stromal cells have been split into two phenotypes present in both samples. The phenotypes determined by the expression of PTEN (Fig. 20 e), with cells expressing the protein shown in orange and those lacking the protein shown in light blue. Each phenotype is formed of two CMPs, one located in the cell cytoplasm and one localised to the nucleus and vesicles. The two CMPs are differentiated by expression of MSH2. The lack of a purely nuclear marker in the stromal cells means that the CMP analysis of these cells is unable to segment the nuclei. On the other hand, P53 has allowed identification of nuclei in the healthy epithelial cells expressing the protein (Fig. 20 c) resulting in a unique CMP shown in dark red in Fig. 20 a. In the epithelial cancer cells, the mutation of MLH1 has resulted in unique CMPs being identified (Fig. 20 f). Similar to the healthy epithelial cells, the expression of P53 (Fig. 20 d) divides the cells into two phenotypes.

It is clear that this kind of analysis can allow for the identification of different cell phenotypes and subcellular compartments that may shed new light on tumour heterogeneity. In the experiment above, CMP analysis was unable to identify the nucleoli and golgi apparatus. This is because the proteins expressed in the nucleoli are also expressed in the nuclei. Similarly, the only protein expressed in the golgi is also expressed weakly in the cytoplasm. If one was interested in identifying these regions, a higher threshold could be set to ignore weak expression. However, great care would need to be taken as the texture of the protein expression may result in holes in the cytoplasmic or nuclear regions.

### Protein network analysis

Kovacheva et al. [36] introduced the DiSWOP framework for analysing multiplex IF data, such as the one simulated by the model described above. The approach analyses cell phenotypes in normal and cancerous colon tissue imaged using the TIS microscope [34]. It involves segmenting the image into cells and determining the cell phenotypes according to their protein-protein dependence profile. Calculating the DiSWOP measure enables identification of protein pairs which have significantly higher/lower co-expression levels in cancerous tissue samples when compared to normal colon tissue. We apply the DiSWOP framework to a set of simulated images. For this purpose, we generated 10 healthy and 10 moderately differentiated cancerous samples at 40 × magnification. From the 10 cancerous samples, 4 had no mutation, 3 had a mutation in the MLH1 gene and 3 had a mutation in the MSH2 gene. The same dataset was also simulated at 20 × magnification to investigate the dependence of the DiSWOP measure on the magnification scale. This experiment was conducted considering only proteins directly linked to MSI, i.e. without simulating the expression of PTEN.

**Fig. 21:**
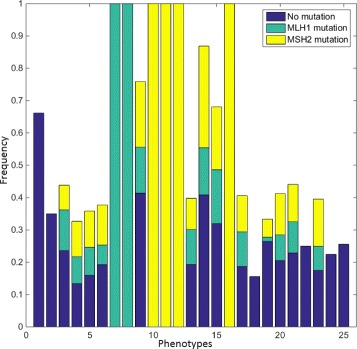
Distribution of phenotypes within cancer samples simulated at 40 × magnification. Phenotypes are shown along the *x*-axis and the fraction of the phenotype that is found within each type of cancer samples is shown along the *y*-axis. Cancer samples without mutation are shown in blue, samples with MLH1 mutation are shown in teal and yellow shows the samples with MSH2 mutation

**Fig. 22:**
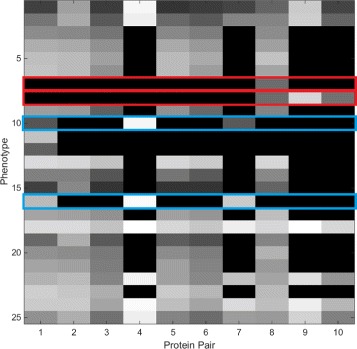
Average protein-protein dependence profiles (PPDPs) for the phenotypes found within healthy and cancerous samples simulated at 40 × magnification. Phenotypes found only in samples with MLH1 mutation are highlighted in red. Similarly, phenotypes found only in samples with MSH2 mutation are highlighted in blue. Numbering of the phenotypes is the same as in Fig. 21. Numbering of the protein pairs is shown in Table 5. Black indicates PPD value of 0, and white shows a PPD value of 1

**Fig. 23:**
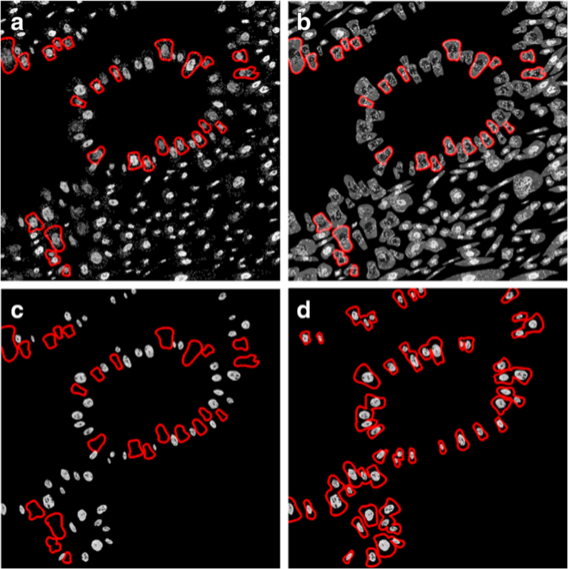
Simulated protein expression in cell phenotypes found only in MLH1 mutated samples. The images show the expression for **a** MSH2, **b** MSH6 and **c**, **d** P53. The red outlines indicate the cells belonging to phenotypes (**a** – **c**) 7 and (**d**) 8

**Fig. 24:**
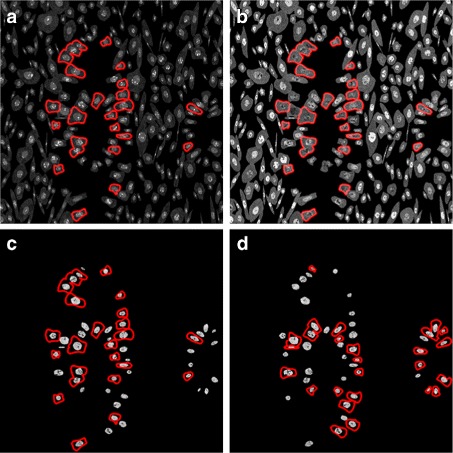
Simulated protein expression in cell phenotypes found only in MSH2 mutated samples. The images show the expression for **a** MLH1, **b** PMS2 and **c**, **d** P53. The red outlines indicate the cells belonging to phenotypes (**a** – **c**) 10 and (**d**) 16

For each of the cells, we calculate the protein-protein dependence profile (PPDP) using the Maximal Information Coefficient (MIC) [37]. The protein pairs are shown in Table 5. The cells are phenotyped using Affinity Propagation [38] according to their PPDP. Distribution of the phenotypes within the cancerous samples simulated at 40 × magnification is shown in Fig. 21. We can see that phenotypes 7 and 8 are found only in samples with MLH1 mutation. Their PPDPs are highlighted in red in Fig. 22. From Fig. 22, we can see that phenotype 7 exhibits non-zero dependence only between MSH2 and MSH6, whereas phenotype 8 also has non-zero dependencies between these two proteins and P53. This can also be observed from the real data. We can see in Fig. 23 that the two phenotypes include all of the epithelial cells, with phenotype 8 including all epithelial cells expressing P53. On the other hand, phenotypes 10, 11, 12 and 16 are found only in samples with MSH2 mutation. Phenotypes 10, and 16 (marked in blue in Fig. 22) show non-zero dependencies between MLH1, PMS2 and P53, splitting the epithelial cells expressing P53 in two phenotypes. These are shown in Fig. 24. This demonstrates that the clustering is able to detect meaningful cell phenotypes, although the real phenotypes could be split into two or more phenotypes found by the algorithm.

**Table 5:** Protein pair numbering

	PMS2	MSH2	MSH6	P53
MLH1	1	2	3	4
PMS2		5	6	7
MSH2			8	9
MSH6				10

**Fig. 25:**
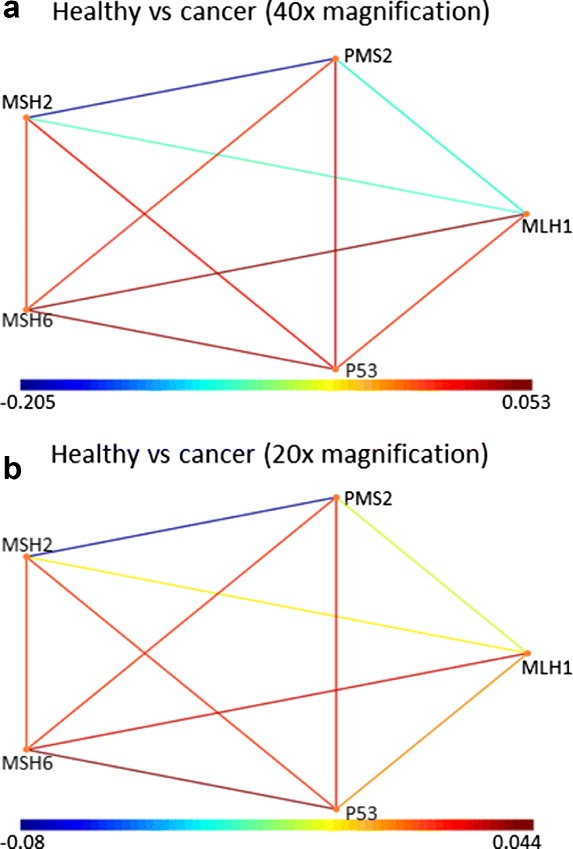
DiSWOP results for the simulated samples at (**a**) 40 × and (**b**) 20 × magnification. Each node represents a protein and each edge colour shows a protein pair with different level of co-expression in the normal and cancer samples. Here, a large positive value (*shown in red*) indicates that the protein pair is more co-dependent in cancer samples, whereas a large negative value (*shown in blue*) means that the protein pair is more active in normal tissue

**Fig. 26:**
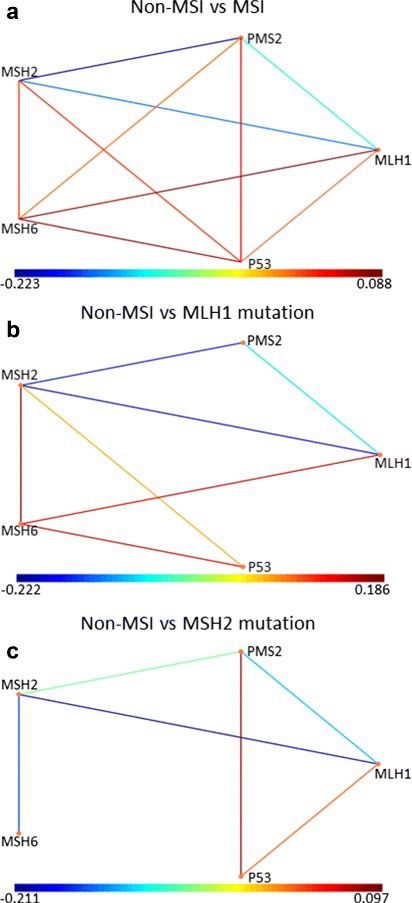
DiSWOP results for comparing MSI and non-MSI sets of the simulated cancer samples at 40 × magnification. Different results are shown when comparing non-MSI samples to (**a**) both mutations, (**b**) MLH1 mutation only, and (**c**) MSH2 mutation only. Each node represents a protein and each edge colour shows a protein pair with different level of co-expression in the normal and cancer samples. Here, a large positive value (*shown in red*) indicates that the protein pair is more co-dependent in the mutated samples, whereas a large negative value (*shown in blue*) means that the protein pair is more active in non-MSI tissue

Once we have obtained the phenotypes, we calculate the DiSWOP measure. We consider the top 3 protein pairs in each phenotype due to their relative significance. The DiSWOP results for the simulated samples at 40 × and 20 × magnification are shown in Fig. 25. We can see that nearly the same results are obtained, demonstrating that the measure is independent of the magnification scale and size of the cells. Figure 25 also shows that DiSWOP is able to detect that the dependences between MLH1, PMS2 and MSH2 are stronger in the healthy samples, suggesting that they are broken in at least some of the cancer samples. However, it is difficult to interpret the results further as within the cancer samples there are a number of non-MSI samples and cells that have the same protein expressions as the healthy samples. To further analyse the simulated data, we considered dividing the cancer samples into three sets depending on the presence of a mutation. We re-run the analysis framework when considering non-MSI samples versus MSI samples with both mutations (Fig. 26 a), and versus each mutation separately (Fig. 26 b and c). When samples with both mutations are considered, the results are very similar to those seen in Fig. 25. This is due to the fact that the mutations cause all of the protein pair interactions to be broken down in some of the samples. However, the negative values again clearly indicate the lack of co-localisation of the MMR proteins. On the other hand, if we consider non-MSI samples versus samples with MLH1 mutation (Fig. 26 b), we can see that, as expected, the interactions of MLH1 and PMS2 are weaker in the MSI sample while MSH6 shows stronger interactions with MSH2, P53 and MLH1. The latter interaction is likely to occur only in the stromal cells which express all proteins. Lastly, we compared non-MSI and MSH2 mutated samples (Fig. 26 c). As would be expected, we observe stronger interactions of MSH2 with other proteins in the non-MSI samples. The mutated samples are characterised by increased co-localisation of P53 and PMS2.

With this set of proteins, it would be easier to simply consider the raw protein expression values. This is because there is no evidence to suggest that the expression patterns of these proteins within the cells change as a result of cancer and this has been reflected in the model. Hence, this experiment aims to demonstrate only how the DiSWOP framework could be used to analyse the synthesised data. However, DiSWOP would provide a significantly greater advantage if the simulated proteins changed their subcellular expression patterns [36]. Proteins that exhibit such changes in localisation could be easily modelled using the framework presented above. These could be proteins with known response to cancer or one could generate random changes in localisation in order to test hypotheses.

## Conclusions

We presented protein expression models to simulate multiplexed IF data of both healthy and cancerous colorectal samples. We investigate how to realisticly simulate the expression of six proteins associated with MSI or tumour suppression, namely MLH1, PMS2, MSH2, MSH6, P53 and PTEN. Following the same method, further proteins of interest could be easily added to the model to increase its usability and study differential co-localisation of proteins. In order to simulate the subcellular location of the proteins, we have developed models for the cell nucleoli, golgi and vesicles, using parameters obtained from real fluorescence data of cells in culture. Comparison between the distribution of various features obtained from the real and synthetic organelles has shown very good agreement. This has included both features that have been used as part of the model input and ones that have not been explicitly considered. The addition of further proteins of interest may require more of the cell organelles to be modelled, such as the cytoskeleton and the endoplasmic reticulum. It would be difficult to represent these using the deformed circle model, so a different approach may need to be developed. We have analysed simulated data using the combinatorial molecular phenotype analysis and have demonstrated that this approach is capable of identifying the different cell phenotypes and subcellular structures in the tissue. Finally, we presented a study of how the DiSWOP framework could be used to analyse the synthetic data. Using the framework to compare the protein co-localisation in MSI versus non-MSI samples was able to detect the presence of mutations. This kind of analysis would be invaluable in detecting changes in subcellular expression patterns resulting from the development of cancer. Proteins that exhibit such changes in localisation could be easily modelled using the framework presented in order to test various hypotheses.

To the best of our knowledge, this is the first model for subcellular expression of multiple proteins in anatomically intact tissue, as opposed to existing models for protein expression within cells in culture. The synthetic data generated using this model could provide an objective way of validating and comparing image analysis methods such as cell-compartment classification methods, frameworks for studying protein co-localisation or protein expression grading.

## Abbreviations

CMP: Combinatorial molecular phenotype
CRA: Colorectal adenocarcinoma
HPA: Human protein atlas
IF: Immunofluorescence
IHC: Immunohistochemistry
MMR: Mismatch repair
MSI: Microsatellite instability
PDF: Probability distribution function
TIS: Toponome imaging system
